# Protocol of changes induced by early Hand-Arm Bimanual Intensive Therapy Including Lower Extremities (e-HABIT-ILE) in pre-school children with bilateral cerebral palsy: a multisite randomized controlled trial

**DOI:** 10.1186/s12883-020-01820-2

**Published:** 2020-06-12

**Authors:** Rodrigo Araneda, Stephane V. Sizonenko, Christopher J. Newman, Mickael Dinomais, Gregoire Le Gal, Daniela Ebner-Karestinos, Julie Paradis, Anne Klöcker, Geoffroy Saussez, Josselin Demas, Rodolphe Bailly, Sandra Bouvier, Emmanuel Nowak, Andrea Guzzetta, Inmaculada Riquelme, Sylvain Brochard, Yannick Bleyenheuft

**Affiliations:** 1 Institute of Neuroscience, Université catholique de Louvain, Avenue Mounier 53 box B1.53.04, 1200 Brussels, Belgium; 2 Division of Child Development and Growth, Department of Pediatrics, University of Geneva, Geneva, Switzerland; 3 Paediatric Neurology and Neurorehabilitation Unit, University Hospital of Lausanne, Lausanne, Switzerland; 4 Département de Médecine Physique et de Réadaptions, CHU Angers-Capucins, Angers, France; 5 Laboratoire Angevin de Recherche en Ingénierie des Systèmes, Université d’Angers, Angers, France; 6 University Hospital of Brest, Brest, France; 7 INSERM CIC 1412, Brest, France; 8 Department of Developmental Neuroscience, IRCCS Fondazione Stella Maris, Pisa, Italy; 9 Haute Ecole Léonard de Vinci, Parnasse-ISEI, Brussels, Belgium; 10 Institut Régional de Formation aux Métiers de Rééducation et de Réadaptation (IFM3R), Nantes, France; 11 Pediatric rehabilitation department, Fondation Ildys, Brest, France; 12 INSERM UMR 1101, LaTIM, Brest, France; 13 Western Britany University, Brest, France; 14 Department of Clinical and Experimental Medicine, University of Pisa, Pisa, Italy; 15 Department of Nursing and Physiotherapy and Research Institute on Health Sciences (UINICS-Idisba), University of the Balearic Islands, Palma de Mallorca, Spain

**Keywords:** Cerebral palsy, Intensive training, Bilateral, Randomized controlled trials, Neuroplasticity, Biomechanical changes

## Abstract

**Background:**

Cerebral palsy (CP), which is the leading cause of motor disability during childhood, can produce sensory and cognitive impairments at different degrees. Most recent therapeutic interventions for these patients have solely focused on upper extremities (UE), although more than 60% of these patients present lower extremities (LE) deficits. Recently, a new therapeutic concept, Hand-arm Bimanual Intensive Therapy Including Lower Extremities (HABIT-ILE), has been proposed, involving the constant stimulation of UE and LE. Based on motor skill learning principles, HABIT-ILE is delivered in a day-camp setting, promoting voluntary movements for several hours per day during 10 consecutive week days. Interestingly, the effects of this intervention in a large scale of youngsters are yet to be observed. This is of interest due to the lack of knowledge on functional, neuroplastic and biomechanical changes in infants with bilateral CP. The aim of this randomized controlled study is to assess the effects of HABIT-ILE adapted for pre-school children with bilateral CP regarding functional, neuroplastic and biomechanical factors.

**Methods:**

This international, multicentric study will include 50 pre-school children with CP from 12 to 60 months of age, comparing the effect of 50 h (2 weeks) of HABIT-ILE versus regular motor activity and/or customary rehabilitation. HABIT-ILE presents structured activities and functional tasks with continuous increase in difficulty while the child evolves. Assessments will be performed at 3 period times: baseline, two weeks later and 3 months later. The primary outcome will be the Gross Motor Function Measure 66. Secondary outcomes will include Both Hands Assessment, Melbourne Assessment-2, Semmes-Weinstein Monofilament Test, algometry assessments, executive function tests, ACTIVLIM-CP questionnaire, Pediatric Evaluation of Disability Inventory (computer adaptative test), Young Children’s Participation and Environment Measure, Measure of the Process of Care, Canadian Occupational Performance Measure, neuroimaging and kinematics.

**Discussion:**

The results of this study should highlight the impact of a motor, intensive, goal-directed therapy (HABIT-ILE) in pre-school children at a functional, neuroplastic and biomechanical level. In addition, this changes could demonstrated the impact of this intervention in the developmental curve of each child, improving functional ability, activity and participation in short-, mid- and long-term.

**Name of the registry:**

Evaluation of Functional, Neuroplastic and Biomechanical Changes Induced by an Intensive, Playful Early-morning Treatment Including Lower Limbs (EARLY-HABIT-ILE) in Preschool Children With Uni and Bilateral Cerebral Palsy (HABIT-ILE).

**Trial registration:**

NCT04017871

**Registration date:**

July 12, 2019.

## Background

Cerebral palsy (CP) is the most common motor disability in pediatrics with a prevalence between 2 to 3.6 out of 1000 live births [1]. Aside from the motor impairments characterizing the abnormal movement patterns and posture, cognitive and sensory function may also be frequently impaired [2, 3]. These impairments depend on the timing, extent and location of the brain lesions, thereafter determining how the whole-brain operates [4, 5]. Most children with cerebral palsy showed a bilateral brain alteration producing bilateral motor impairment [6–8]. Until now, in clinical practice, most therapies used have shown to be ineffective, based mainly on passive guided movement or passive stretching [9–11]. Over the last 20 years, rehabilitation programs based on scientific evidence have been created aiming to decrease the functional consequences of motor disabilities and consequently impacting positively people’s activity and participation [12, 13]. Among these programs, specifically in school-age children with CP, those promoting high intensity, voluntary movements, increase in difficulty and aiming to functional goals, have proven efficiency in improving motor function [11]. However, little is known about the efficiency of these interventions, and the mechanisms associated, in pre-school children despite evidence showing major brain growth and development occurring in the course of the first 2 years of life [13].

Up to present, intensive therapies in young children have been focused mainly in children with unilateral CP [11] showing encouraging results. Most of these studies evaluated modified forms of constraint-induced movement therapy (CIMT). An adapted baby-CIMT procedure for infants (< 12 months) was proposed by Eliasson and her colleagues [14]. Another adapted CIMT trial was performed in children between 2 and 3 years of age [15, 16]. In addition, 2 research teams tested the effectiveness of CIMT in children with unilateral CP between the ages of 1 and 6 years [17, 18]. Likewise, Ferre et al. (2015), proposed an intensive bimanual therapy in children aged from 2 to 4 years [19]. The application of an intensive therapy in young children is in agreement with the evidence observed in animal models where, after an early brain lesion, different process such as the neurogenesis, axonal growth, synaptogenesis or the myelination can be impacted secondary to the inflammation [20–23]. There is animal model evidence of partial reversal of these alterations after early motor interventions based in motor skill learning when performed at a optimal developmental opportunity window [24–27]. This supports the use of these interventions in children with CP, probably encountering similar neuroplastic changes traduced in functional abilities improvements.

Hand-arm Bimanual Intensive Therapy Including Lower Extremity (HABIT-ILE) [28] is an intensive therapy based on the principles of motor skill learning that involves the constant stimulation of upper extremities (UE) as well as lower extremities (LE) and posture. In school age children with bilateral CP this intensive training has shown improvements in UE and LE motor function [29] after 84 h of therapy delivered in 6,5 h per day during 13 consecutive days. Recently, a pilot program for pre-school children (*n* = 10) with unilateral CP (“early HABIT-ILE”) has shown interesting results [30]. In this pilot, HABIT-ILE was adapted in dosage, being performed during 5 h per day during 10 consecutive week days. Despite this difference, early HABIT-ILE showed significant improvements in the more and the less affected hand of children, as well as in gross motor function [30]. Nevertheless, whether this intervention can be performed in children with a bilateral CP of this age is unknown. Moreover, changes at the functional, neuroplastic and biomechanical level have never been assessed in young infants with bilateral CP. Therefore, the aim of this randomized control trial (RCT) is to assess, in pre-school children with bilateral CP, the impact of early HABIT-ILE (e-HABIT-ILE) on functional, neuroplastic and biomechanical parameters.

### Aims and hypotheses

This multicentric RCT was designed to determine the effect of e-HABIT-ILE following 2 weeks of therapy and at three months follow up. In addition, the e-HABIT-ILE effect on neuroplasticity and movement characteristics of the UE and LE will be tested exclusively at three months.

### Primary aim

The main objective of this RCT is to evaluate whether two weeks of the e-HABIT-ILE program is more effective at mid-term (three month) than usual care (control group) in improving gross motor function (assessed by the Gross Motor Function Measure (GMFM-66)) in 50 preschool children, aged 1 to 4 years, with bilateral cerebral palsy.

### Primary hypothesis

*e-HABIT-ILE will induce, in children with bilateral CP, greater improvements in gross motor function than usual motor activity including usual rehabilitation, at three months.*

### Secondary aims and hypotheses

- Aim: To establish the e-HABIT-ILE effectiveness on bimanual activity performance.

*Hypothesis: BOHA scores will be higher for e-HABIT-ILE group than control group.*

- Aim: To establish the e-HABIT-ILE effectiveness on unimanual performance of the more affected and less affected hand of children.

*Hypothesis: Melbourne Assessment-2 scores will be higher for e-HABIT-ILE group than control group.*

- Aim: To establish the e-HABIT-ILE effectiveness on tactile threshold for the more affected and less affected hands.

*Hypothesis: Tactile thresholds will be lower (improved tactile sensation using the Semmes-Weinstein Monofilament test) for e-HABIT-ILE group compared to control group.*

- Aim: To establish the e-HABIT-ILE effectiveness on pressure pain threshold for the more affected and less affected hands.

*Hypothesis: Pressure threshold will be higher (lower pressure sensitivity using a pressure algometer) for e-HABIT-ILE group compare to control group.*

- Aim: To establish the e-HABIT-ILE effectiveness on executive function.

*Hypothesis: Higher scores on working memory and inhibitory control tests will obtain the e-HABIT-ILE group compared to the control group.*

- Aim: To establish the e-HABIT-ILE effectiveness on global activity performance in daily life activities.

*Hypothesis: Higher scores on the ACTIVLIM-CP and the Pediatric Evaluation of Disability Inventory Computer Adaptive Test (PEDI-CAT) will obtain the e-HABIT-ILE group compared to the control group.*

- Aim: To establish the e-HABIT-ILE effectiveness on social participation.

*Hypothesis: Higher scores on the Young Children’s Participation and Environment Measure (YC-PEM) will obtain the e-HABIT-ILE group compare to the control group.*

- Aim: To establish the e-HABIT-ILE effectiveness on health services perception.

*Hypothesis: Higher scores on the Measure of the Process of Care (MPOC-20) will obtain the e-HABIT-ILE group compared to the control group.*

- Aim: To establish the e-HABIT-ILE effectiveness on parents-determined functional goals.

*Hypothesis: Higher scores on the Canadian Occupational Performance Measure (COPM) will obtain the e-HABIT-ILE group compared to the control group.*

- Aim: To establish the e-HABIT-ILE effectiveness in generating neuroplastic changes at grey and white matter level together with brain connectivity.

*Hypothesis: Advanced brain imaging techniques will show greater changes in grey and white matter, and in connectivity for the e-HABIT-ILE group compare to the control group.*

- Aim: To establish the e-HABIT-ILE effectiveness in generating movement characteristics’ changes on upper and lower extremities.

*Hypothesis: Greater improvements in kinematic parameters for upper and lower extremities movements will obtained the e-HABIT-ILE group compared to the control group.*

## Methods

### Ethics

Full ethical approval has been obtained in each of the centers involved in this study: Belgium (B403201316810), Italy (244/2019) and France (29BRC19.0050/N2019-A01173–54). A straight and comprehensive information document will be given to parents who will be asked to sign a consent form if they comply to their child’s participation. All data collected for the purposes of this study will be treated anonymously. This study has been registered in the Clinical Trial Registry (NCT04017871).

### Patient and public involvement

The opinion of a French Association of people with cerebral palsy was considered for the design of this protocol.

### Study design

**Fig. 1:**
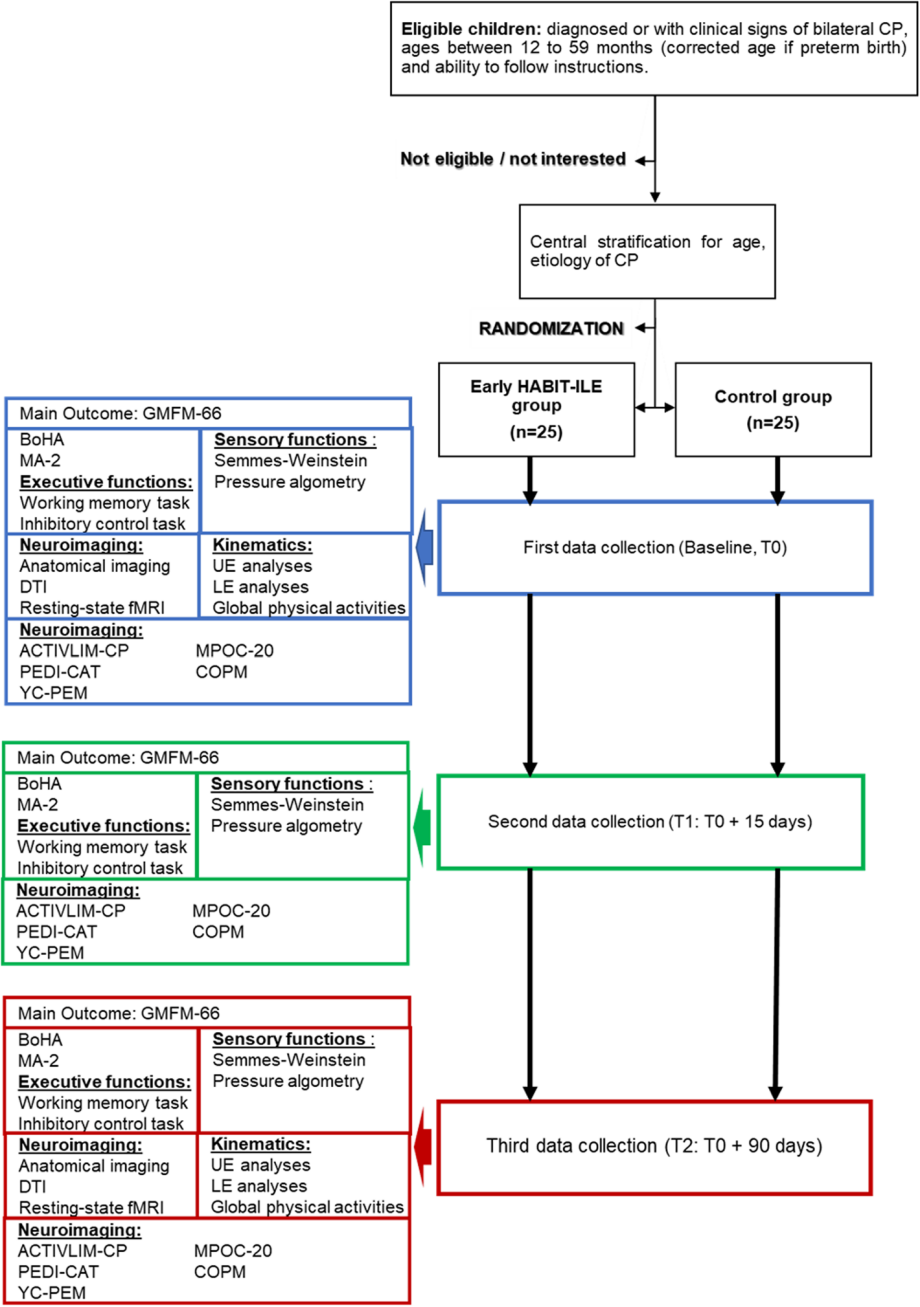
CONSORT Flowchart. RCT, randomized controlled trial; CP, cerebral palsy; GMFCS, gross motor function classification system; BOHA, both hands assessment; GMFM-66, gross motor function measure (66 items); MA-2, Melbourne assessment 2; PEDI-CAT, pediatric evaluation of disability inventory computer adaptive test; YC-PEM, young children’s participation and environment measure; MPOC-20, measure of the process of care (20 items); COPM, Canadian occupational performance measure; DTI, diffusion tensor imaging; fMRI, functional magnetic resonance imaging; UE, upper extremities; LE, lower extremities

We will implement a multi-center RCT in three European countries: Belgium (Brussels), Italy (Pisa) and France (Brest and Angers). The study will compare the effect of two weeks of e-HABIT-ILE to that of usual motor activity, including usual rehabilitation. Children will be assessed at 3 period times: baseline (T0), two weeks after baseline (T1) and 3 months after baseline (T2). CONSORT guidelines will be followed when reporting this RCT (See Fig. 1).

### Recruitment

#### Participants

For this study, fifty pre-school children aged between 1 to 4 years, with bilateral CP will be included. Children will be recruited, in Belgium, from the CP center of Saint-Luc University Hospital; from the Centre for Innovative Therapies in CP of the Stella Maris Scientific Institute in Pisa, Italy; and from the Brest University Hospital Center, the Angers University Hospital Center and Les Capucins Specialized Rehabilitation Center in France. Furthermore, for those children not attending any of these centers, spontaneous applications from parents will be considered.

#### Inclusion criteria

Children will be considered for eligibility if they are aged 12 to 59 months old (corrected age if preterm birth), have a confirmed diagnosis or presumption of bilateral CP, either spastic or dyskinetic and are able to follow instructions and accomplished all necessary age-suited assessments.

#### Exclusion criteria

Children will not be considered to participate if they present uncontrolled seizures, had botulinum toxin injections or orthopedic surgery within the past six months, or if such interventions are planned during the study period, have severe visual or cognitive impairments interfering with treatment and or assessments, have any typical contraindication for magnetic resonance imagery (MRI), or inability/refusal consent of parents for participation of their child.

### Randomization process

Using a blind centralized electronic distribution system, children will be assigned either to a control or a treatment group. Before the first assessment and collection of informed consent, children will be paired regarding age at inclusion (1/2/3/4 years), etiology of CP (brain malformation/grey matter injury/periventricular white matter injury),

### Sample size

The sample size calculation was based on the mean improvement in the GMFM-66 in the study by Bleyenheuft et al. (2017) [29]. In that study of school-aged children, the GMFM-66 increased by 7 logits in the intervention group and by 2 logits in the control group. The duration of the intervention (2 weeks) and the follow up period (3 months) were the same as in the present study. Considering the differences between the samples and the potential variability of our target population (pre-school children), we estimate that the standard deviation of change could reach a maximum of 5 logits. We thus hypothesized that there would be a minimum difference of 5 logits between groups, with a standard deviation of 5 logits, an α of 0.05 and a 1-β of 0.9. Consequently, 23 participants are needed per group (total of 46). Considering potential drop-outs, 50 participants will be included.

### Current status of the protocol

This multi-center protocol is currently between the process of recruitment and initiating the period of intervention.

### Blinding procedure

External accredited/experienced raters, blind to group allocation and timing of assessment, will score videos of the main outcome of this multicentric RCT (GMFM-66), as well as two secondary outcomes (BoHA and MA2). Analyses will be performed using allocation codes. Conversely, kinematic and neuroimaging data analyses will be conducted by an experienced rater, blind to group allocation and timing assessment. Data will be anonymized and stored within each site and will be delivered for analysis after the RCT ends.

### Study interventions

#### HABIT-ILE procedure

HABIT-ILE [28, 29, 31] is an intensive intervention based on the principles of motor skill learning. Is delivered in a day-camp context, promoting structured activities with increasing motor difficulty, as well as functional tasks. These activities involve bimanual use, concomitantly demanding postural and/or LE activities. This program was adapted following the experience of our pilot study [30] and the know-how developed in school-age children with bilateral CP [28, 29].

For pre-school children, HABIT-ILE is presented as a fun, playgroup-like environment in which activities are always ludic. All activities will be determined by the supervisors of e-HABIT-ILE regarding baseline assessments (T0) and individualized functional goals (e.g. walking with walker, learning to crawl, playing standing with one hand in support, etc.). These last will be defined with the parents at T0. During the 10 consecutive week days of intervention, each activity will increase in difficulty, considering in this shaping the constant use of both hands, the LE and the postural component. The level of difficulty will be individualized for each child but will always challenge the use of upper and lower extremities (from simple passive stabilizing activities towards active use in UE, and from stable/lower positions on the ground or bench towards unstable or higher positions as standing in LE).

#### Implementation of e-HABIT-ILE

e-HABIT-ILE will be delivered to a group of six to nine children simultaneously, with activities designed for a single child or in group. Every child will be accompanied at all times by, at least, 2 adults: one therapist (PT or OT) and one student therapist. They will be the child “playmates”. A group of at least 3 experienced e-HABIT-ILE therapists will supervise the therapy to warrant that the content and structure of the activities are carried out respecting the HABIT-ILE principles. A total of 5 h per day (3 h in the morning and 2 h in the afternoon, with 2,5 h of rest in between), during 10 days, will allow us to complete 50 h of therapy for this study. This dosage was determined according to the usual amount of motor activity experienced by pre-school children and infants [32], current scientific evidence on dosage [33, 34] and our experience regarding feasibility in this age group [30]. Regardless of allocation, all children will benefit from the therapy.

#### Control procedure

Those children allocated to the control group will continue with their regular/customary care routine, including usual rehabilitation. Pre-school children are reported to occupy about 7.6 h per day in motor activities [32]. But if we consider activities performed at home such as dressing, eating or bathing, motor activities performed in daycare account for around 5 hours [32]. To document the content of these activities, a logbook will be filled by the caregiver. Moreover, wrist sensors will register the amount of activity during 5 days. This recording will be performed both in the control and treatment group.

#### Data monitoring committee (DMC)

A research member of each site will participate of the DMC if needed, evaluating and handling any adverse incidents throughout the study at 6 months intervals.

### Outcomes

#### Primary outcomes

The primary outcome measure will be the difference between groups (e-HABIT-ILE and control) for changes in scores (T2-T0) in Gross Motor Function Measure (GMFM–66, measured in % of logits) [35]. This tool evaluates changes in gross motor function over time or following an intervention in children with CP using Rasch analysis. The items cover a large spectrum of activities ranging from lying and rolling to walking, running and jumping. Its responsiveness [36], reliability and validity have been demonstrated in children with CP [37, 38] including infants and toddlers [39].

#### Secondary outcomes

##### Functional assessments (assessed at T0, T1 and T2)

To evaluate bimanual activity performance and level of upper extremity asymmetry, the Both Hands Assessment [40] (BoHA) will be used. This is a valid test for children with mild to moderate bilateral manual abilities impairments.

To evaluate unilateral upper limb function, the Melbourne Assessment-2 [41] (MA2) will be used. This is a valid and reliable test for quality of upper limb movements in children with neurological disorders.

To evaluate tactile threshold, the Semmes-Weinstein Monofilament Test will be used. This is a reliable and reproducible test [42, 43].

To evaluate pressure threshold a standardized algometer will be used. This tool is well-tolerated by young children [42], with an excellent intra-rater and inter-rater reliability.

As described by Gottwald et al., (2016) [44]in younger children, executive functions will be assessed through working memory and inhibitory control.

To assessed global activity performance in activities of daily living, the ACTIVLIM-CP [45] questionnaire will be used. This is a reliable and valid tool assessing the use of UE and LE in everyday life for children with CP.

To assess functional skills in mobility and daily activities, the Pediatric Evaluation of Disability Inventory, computer adaptive test (PEDI-CAT) [46] will be used. This questionnaire has shown to be sensitive when used in CP population [47].

To assess social participation, the Young Children’s Participation and Environment Measure [48] (YC-PEM) will be used. This questionnaire has shown good intra-rater and inter-rater reliability.

To assess the parents’ perception about the degree of family-focused approach their family receives, the Measure of the Process of Care [49] (MPOC-20) questionnaire will be used. This is a valid and reliable self-report survey.

To define therapeutic goals, the Canadian Occupational Performance Measure [50] (COPM) will be used. This semi-structured interview defines and quantifies children’s performance regarding these goals, as well as parents’ satisfaction regarding their accomplishment.

##### Neuroimaging (assessed at T0 and T2)

Compatible scanners and standardized acquisition parameters, allowing a pooled posterior analysis, will be used at the 4 sites: Saint-Luc University Hospital in Brussels, Scientific Institute for Research, Hospitalization and Health Care Stella Maris in Pisa, Morvan Regional University Hospital Centre of Brest and at the University Hospital of Angers. All children (and one parent when applicable) will be scrutinized before the exam, ensuring no contraindications to perform the MRI. The scanner procedure will be under anesthesia or during spontaneous sleep considering the parents’ preference. Children will endure a standardized gradual familiarization program aiming to become comfortable in the MRI environment. The familiarization will include the use of ear-plugs to sleep, particular observation of the child’s sleep routine and feeding immediately before the scan in spontaneous sleep process [51, 52]. The total time of MRI is estimated to 20 min.

Analyses of morphometry concentrated on structural neuroplastic changes comprising cortical thickness, cortical folding and white matter fiber shape will be performed using high-resolution 3D T1-weighted MRI.

Corticospinal tract organization and any changes will be determined first through fiber tracking using diffusion tensor imaging. Fiber quality changes of the tract will be assessed by analyzing fractional anisotropy, main diffusivity and tract volume [53, 54].

Topological properties of networks on the whole-brain will be determined using fMRI by analyzing connectivity using graph theory and network analysis. Motor network properties will also be assessed [55, 56].

##### Movement parameters (assessed at T0 and T2)

All movement assessments will be performed at the corresponding 3D motion laboratories of the consortium using the same standardized acquisition protocol and optoelectronic system. Within a total estimated time < 45 min, all children will undergo UE analyses. In addition, the children with independent walking or necessitating minimum assistance, will also undergo LE analyses. We expect to accomplish a 60% of entire acquisitions based on the Brest team expertise and recent evidence on 10-month old infants [57].

To assess UE 3D motion analyses, an adapted version of previous procedures [58] will be used as protocol. Anatomical landmarks on upper limb will include markers on the thorax, acromion, arm, forearm, hand and fingers. For the tasks, children will be positioned in a standardized chair facing a Table. A total of three different reach-to-grasp tasks will be presented. The Arm Profile Score [59] will be used to assessed kinematic and spatiotemporal parameters. In addition, electromyographic signals in four muscles (long head of the triceps brachii, short head of the biceps brachii, pronator teres and quadratus) will be analyzed following Sarcher et al. methods [60] as well as guidelines of the European project “Surface Electromyography for the Non-Invasive Assessment of Muscles”(SENIAM) [61]. The EMG-Profile Score for UE motion [62] will be used to assessed muscle activation indices.

To assess gait 3D motion, sixteen anatomical landmarks will be highlighted with reflective markers following Davis’ protocol [63]. Barefoot, children will be asked to walk unassisted or with minimum assistance down a 10-m-long path, registering a minimum of ten trials. Kinematic and spatio-temporal parameters will be analyzed. In addition, electromyographic signals in five muscles (rectus femoris, vastus lateralis, medial hamstrings, tibialis anterior and gastrocnemius-soleus) will be analyzed using a 16-channel electromyography system following the SENIAM guidelines [61].

To assess global physical activity, wrist sensors [64, 65] will be used to measure active and resting states, including periods of intensity, posture and gait using bar-code parameters. Wrist sensors have the advantage of having automatic calibration and simple positioning. Total time spent in movement (measured in percentage), such as crawling, walking or running during either the HABIT-ILE or the control period, will be considered as the main outcome.

### Statistical analysis

Primary outcome changes (GMFM-66) will be assessed using analysis of covariance (ANCOVA) between groups, including adjustment for baseline measurements following Vickers’s recomendations [66]. Secondary outcomes changes will be also analyzed with ANCOVA measurements (or non-parametric equivalent when normality and homoscedasticity are not met). For exploratory reasons, other subgroup characteristics (such as age) will also be analyzed.

## Discussion

This protocol targets pre-school age children with bilateral CP which is the most prevalent topographic presentation of CP. In addition, the evidence about the effective therapies for this population is very limited. For this reason, we expect that if e-HABIT-ILE show important improvements in the different outcomes (functional, neuroplastic and biomechanical), we could impact on each child’s developmental curve, which would in turn impact the three domains of the International Classification of Functioning, Disability and Health for Children and Youth [67] at a short, mid- and long-terms.

All results emanating from this project will be published in peer-reviewed publications as well as international and local conventions. We expect an international impact in case of positive outcomes and effectiveness of e-HABIT-ILE, possibly changing the vision of these children’s care and, moreover, decreasing the economic load on the health system regarding this population.

## Data Availability

Not Applicable.

## Abbreviations

BOHA: Both Hands Assessment
ANCOVA: Analysis of covariance
CIMT: Constraint-induced movement therapy
COPM: Canadian Occupational Performance Measure
CP: Cerebral Palsy
DMC: Data Monitoring Committee
GMFM-66: Gross Motor Function Measure
HABIT-ILE: Hand-arm Bimanual Intensive Therapy Including Lower Extremities
LE: Lower Extremities
MA2: Melbourne Assessment-2
MPOC-20: Measure of the Process of Care
PEDI: Pediatric Evaluation of Disability Inventory
RCT: Randomized controlled trials
UE: Upper Extremities
YC-PEM: Young Children’s Participation and Environment Measure
